# Prevalence, Antimicrobial Susceptibility Pattern, and Associated Factors of Urinary Tract Infections among Adult Diabetic Patients at Metu Karl Heinz Referral Hospital, Southwest Ethiopia

**DOI:** 10.1155/2018/7591259

**Published:** 2018-11-01

**Authors:** Tesfaye Gutema, Fitsum Weldegebreal, Dadi Marami, Zelalem Teklemariam

**Affiliations:** ^1^Medical Microbiology Unit, Metu Karl Heinz Referral Hospital, Metu, Ethiopia; ^2^Department of Medical Laboratory Sciences, College of Health and Medical Sciences, Haramaya University, Harar, Ethiopia

## Abstract

Urinary tract infection causes considerable morbidity in diabetic patients and if complicated, can cause severe renal damage and life-threatening infections. The escalating antimicrobial resistance rate among bacteria over the past years is another concern in the treatment of urinary tract infections. This study investigated the prevalence, antimicrobial susceptibility pattern of the isolates and associated factors of urinary tract infection among adult diabetic patients attending Metu Karl Heinz Referral Hospital, Southwest Ethiopia. An institutional-based cross-sectional study was conducted among 233 adult diabetic patients selected using simple random sampling technique. Data were collected using a structured questionnaire. Clean-catch midstream urine samples were investigated for the presence of pathogenic bacteria and their antimicrobial susceptibility pattern using recommended culture methods. Data were entered, cleaned, and analyzed using the Statistical Program for Social Sciences version 21.0. Statistical significance was set at a *p*-value < 0.05. The prevalence of urinary tract infection was 16.7% (95%, CI: 12.0, 21.5). The predominant isolates were *Escherichia coli* (25.6%) and *Klebsiella* spp. (20.5%). *E. coli* isolates showed higher sensitivity to ceftriaxone (80%), ciprofloxacin (70%), and gentamycin (70%), but resistant to tetracycline (60%). *Staphylococcus aureus* was sensitive to amoxicillin-clavulanic acid (85.7%), and gentamycin (57.1%), while resistant to tetracycline (85.7%), nitrofurantoin (85.7%), and ampicillin (71.4%). The odds of developing urinary tract infections were significantly higher in diabetic females (AOR: 3.56, 95% CI: 1.44, 8.76), those who were not able to read and write (AOR: 2.55, 95% CI: 1.19, 5.49) and those with a history of urinary tract infection (AOR: 2.31, 95% CI: 1.09, 4.90) compared with their counterparts. In this study, the prevalence of urinary tract infection among diabetic patients was relatively comparable with the previous studies conducted in Ethiopia. Management of urinary tract infection in diabetic patients should be supported with culture and antimicrobial susceptibility testing.

## 1. Introduction

Diabetes mellitus (DM) is a group of metabolic disorder characterized by hyperglycemia resulting from defects in insulin secretion, insulin action, or both [1]. DM has become a serious public health threat in both developed and developing countries, affecting more than 366 million people, and the number is expected to rise to 552 million by 2030 [2]. International Diabetes Federation estimated that 10.8 million people have DM in sub-Saharan Africa in 2006, and this would rise to 18.7 million by 2025 [3].

Diabetes mellitus has long been considered to be a predisposing factor for urinary tract infection (UTI) because of sugar in urine, which serves as media for growth of bacteria [4, 5]. The colonized urinary tract can also accelerate the prolonged release of bacteria with an increased risk of complications of the urinary system, ranging from dysuria (pain or burning sensation during urination) to the organ damage and sometimes even death [6, 7]. The most common bacteria associated with UTI in diabetics are *Escherichia coli*, *Proteus* spp., *Klebsiella* spp., *Pseudomonas aeruginosa*, *Enterococcus* spp., *Staphylococcus aureus*, and coagulase-negative staphylococci (CoNS) [7, 8].

The successful treatment of UTI in diabetic patients depends on the proper identification of the bacteria responsible and the selection of effective antimicrobial agents against them [9]. The problem is particularly exacerbated in low-income countries due to uncontrolled antibiotic use, a high prevalence of fake and spurious drugs of questionable quality in circulation, and lack of infection prevention [10].

Few pocket studies conducted among diabetic patients in different parts of Ethiopia reported a prevalence of UTI ranging from 10.9% to 17.8% with a higher rate of antimicrobial resistance [11–13], indicating the continuing challenge of UTI. The present study was undertaken to assess the prevalence of UTIs, antimicrobial susceptibility pattern of bacterial isolates, and associated factors among adult diabetic patients attending Metu Karl Heinz Referral Hospital, Southwest Ethiopia.

## 2. Materials and Methods

### 2.1. Study Setting

The study was conducted at Metu Karl Heinz Referral Hospital, Southwest Ethiopia from January 2018 to March 2018. Metu is a capital town of Ilubabor Zone, Oromia Regional State. It is located at 600 km from Addis Ababa, Ethiopia. The hospital serves as a teaching and health care providing center for the region. The diabetic treatment center of the hospital provides health care services for about 3127 diabetic patients (source: Hospital Annual Report of 2017).

### 2.2. Study Design and Population

An institutional-based cross-sectional quantitative study was conducted among diabetic patients aged ≥ 15 years who visited the diabetic clinic of the Karl Heinz Referral Hospital. Diabetic patients who used antimicrobial for two weeks before and during data collection, patients admitted for more than 48 hours, and pregnant women were excluded from the study.

### 2.3. Sample Size and Sampling Technique

The sample size was determined using a single population proportion formula by considering the prevalence of UTI among diabetic patients attending Nekemte Referral Hospital, Ethiopia (16.5%) [13], 95% confidence interval, and a 5% margin of error. The final sample size including a 10% non-response rate was 233. Study participants were selected using a simple random sampling technique (lottery method). A complete list of diabetic patients obtained from the hospital diabetic clinic medical record was used as a sampling frame.

### 2.4. Data and Specimen Collection Methods

Socio-demographic characteristics, associated factors and clinical data were collected by two trained nurses using a structured questionnaire developed from different kinds of literature [12, 14, 15]. Each diabetic patient was instructed how to collect a clean-catch midstream urine specimen. Accordingly, about 10 to 15 ml of urine specimen were collected in a labeled, leak proof, and sterile containers. The specimens were stored at 4°C within 30 minutes of collection, and transported under aseptic technique to Nekemte Health Research and Regional Laboratory, Ethiopia. Culture and antimicrobial susceptibility testing were performed by two medical microbiologists. In case of disagreement, researchers were involved in making the decision.

### 2.5. Bacterial Isolation and Identification

Each urine sample was streaked on a freshly prepared differential and selective culture media such as cysteine-lactose-electrolyte-deficient agar, MacConkey agar, mannitol salt agar, eosin methylene blue, and 5% blood agar plates (Oxoid, Ltd, UK) using a calibrated wire loop delivering 0.002 ml of urine. After overnight incubation at 37°C, the plates were inspected for growth and colony characteristics. The incubation was extended up to 48 hours for slow-growing strains. Bacterial colonies differing in size, shape, and color were selected from these plates and separately subcultured in different biochemical tests, which include motility, Gram's reaction, indole tests, methyl red, Voges–Proskauer, citrate utilization, utilization of carbohydrates (such as glucose, sucrose, mannitol, lactose, and fructose), oxidase, catalase, coagulase, and starch hydrolysis test for further characterization and identification [16]. A culture was considered significant for UTI if a single bacterium was recovered at a concentration of ≥10^5^ colony-forming unit per milliliter of urine.

### 2.6. Antimicrobial Susceptibility Testing

An antimicrobial susceptibility profile of each bacterial isolate was determined using the modified Kirby–Bauer disk diffusion method. In brief, 3–5 pure colonies were transferred to a tube containing 5 ml of sterile normal saline (0.85% NaCl) and mixed gently until it forms a homogeneous suspension equivalent to 0.5 McFarland standard. A sterile cotton swab was dipped into the suspension and lawn uniformly over the entire surface of Mueller-Hinton agar (Oxoid, Ltd, UK). Eight Oxoid antimicrobial disks, including amoxicillin-clavulanic acid (30 *μ*g), ampicillin (10 *μ*g), ceftriaxone (30 *μ*g), gentamycin (10 *μ*g), ciprofloxacin (5 *μ*g), norfloxacin (10 *μ*g), nitrofurantoin (50 *μ*g), and tetracycline (25 *μ*g) were placed onto inoculated plates. After overnight incubation at 37°C, the zone of inhibition was measured to the nearest millimeter using a digital caliper. The isolate was classified as sensitive, intermediate sensitive, or resistant based on the Clinical Laboratory Standards Institute (CLSI) criteria [17].

### 2.7. Quality Control

A structured questionnaire was initially prepared in English, translated into the local languages (Amharic and Afan Oromo) and back to English by language experts to maintain its consistency. A questionnaire was pretested on 5% adult diabetic patients attending diabetic center of Bedelle Hospital, Ethiopia prior to the actual data collection, and correction were made based on the pretest feedback. Data collectors were trained for three days (two days before and one day after the pretest) on data collection methods, urine sample collection procedure, sample processing, colony characterization, and antimicrobial susceptibility reading. *E. coli* (ATCC® 25922), *P. aeruginosa* (ATCC® 27853), *Proteus vulgaris* (ATCC® 8427), and *S. aureus* (ATCC® 25923) reference strains were used to check the performance, quality of culture media, and antimicrobial disks.

### 2.8. Methods of Data Analysis

Data were coded, verified, and entered into Epi-Info version 3.5.1 (CDC, Atlanta, GA, USA) and exported into the Statistical Package for Social Sciences software version 21.0 (SPSS Inc, Chicago, IL) for analysis. Descriptive statistics were used to summarize the findings. Bivariate and multivariate logistic regression analyses were used to assess factors associated with UTI. Variables that had a *p* value < 0.25 in the bivariate logistic regression model were considered for a multivariate logistic regression to determine the predictors of UTIs. Those variables with a *p* value < 0.05 at 95% confidence interval were considered statistically significant.

### 2.9. Ethical Consideration

The study was ethically approved by the Institutional Health Research Ethics Review Committee of the College of Health and Medical Sciences, Haramaya University. The study participants were enrolled after written informed consent/assent was obtained. All infected patients were treated based on their laboratory result.

## 3. Results

### 3.1. Participant Characteristics

A total of 233 participants was included in this study. The majority of participants were females (54.1%); with a female to male ratio of 1:0.85. The mean age of participants was 44 years (±15.6 standard deviation). A large proportion (24.9%) of the participants was found to be in the age group between 35 and 44 years. The majority (30.9%) of the study participants had a tertiary level education. Among 233 participants, 40.3% were employed either in governmental or non-governmental organizations (Table 1).

### 3.2. Prevalence of Urinary Tract Infections

The overall prevalence of UTI was 16.7% (95%, CI: 12.0, 21.5). Gram-negative bacteria (64.1%) were the predominant isolates. The most prevalent bacteria isolates were *E. coli* (25.6%) followed by *Klebsiella* spp. (20.5%), *S. aureus* (17.9%), and CoNS (15.4%) (Table 2). In five urine specimens, more than one species of bacteria were isolated. Of these, two bacterial spp. were isolated from 3 specimens and 2 bacteria spp. from 2 specimens.

### 3.3. Antimicrobial Susceptibility Pattern

Gram-negative bacteria were sensitive to ceftriaxone (84%), gentamycin (68%), and amoxicillin-clavulanic acid (64%), but resistant to tetracycline (68%) and norfloxacin (52%). Of Gram-negative isolates, *E. coli* showed higher sensitivity to ceftriaxone (80%), ciprofloxacin (70%), and gentamycin (70%), while it was resistant to tetracycline (60%). *Klebsiella* spp. showed higher sensitivity to ceftriaxone (87.5%) and gentamycin (87.5%), but resistant to tetracycline (75%). *Proteus* spp. were showed higher sensitivity to amoxicillin-clavulanic acid (100%) and ceftriaxone (100%), but were resistant to ciprofloxacin (66.7%), tetracycline (66.7%), gentamycin (66.7%), and norfloxacin (66.7%). *P. aeruginosa* isolates showed highest resistance (100%) to amoxicillin-clavulanic acid, ciprofloxacin, tetracycline, nitrofurantoin, and norfloxacin (Table 3).

Gram-positive bacteria isolates showed a higher level of sensitivity to amoxicillin-clavulanic acid (92.8%) followed by gentamycin (71.4%), but were resistant to tetracycline (71.4%) and nitrofurantoin (64.3%). Of Gram-positive bacteria, *S. aureus* demonstrated a considerable degree of sensitivity against amoxicillin-clavulanic acid (85.7%) and gentamycin (57.1%), but resistance to tetracycline (85.7%), nitrofurantoin (85.7%), and ampicillin (71.4%) (Table 4).

### 3.4. Factors Associated with Urinary Tract Infections

In bivariate logistic regression analysis, variables such as sex, education, history of UTI, current symptoms of UTI, type of diabetes, and history of glucosuria were statistically associated with UTI at a *p* value less than 0.25 and were considered as candidates for multivariate logistic regression. In multivariate logistic regression analysis, the odds of UTI were 3.6 times higher among diabetic females than males (AOR: 3.56; 95% CI: 1.44, 8.76). Diabetic patients who were not able to read and write had 2.6 times the odds of UTI than those who were able to read and write (AOR: 2.55; 95% CI: 1.19, 5.49). The odds of being infected with UTI were significantly higher among diabetic patients who had a history of UTI than those without a history of UTI (AOR: 2.31; 95% CI: 1.09, 4.90) (Table 5).

## 4. Discussion

The overall prevalence of UTI in this study was found to be 16.7% (95%, CI: 12.0, 21.5). This was relatively comparable with the studies conducted in Nekemte, Ethiopia (16.5%) [13], Gondar, Ethiopia (17.8%) [11], Mbarara, Uganda (13.3%) [15], and Dar Es Salaam, Tanzania (13.7%) [18], but lower compared with the previous studies conducted in Janakpur, Nepal (50.7%) [19], and Pashar, Pakistan (51%) [20]. Increased occurrence of UTI among diabetic patients might be due to decreased antibacterial activity, defects in neutrophil function, enough availability of protein, and increased adherence to uroepithelial cells [16, 19].

The most frequent bacteria isolate in this study was *E. coli* (25.6%). This was consistent with studies conducted in Dar Es Salaam, Tanzania (39%) [18], Mbarara, Uganda (50%) [15], and Tehran, Iran (43.8%) [7], but it contradicted with a study finding reported from Nekemte, Ethiopia, in which *S. aureus* and CoNS (24.2%) were most frequently isolated [13]. The higher incidence of *E. coli* could be attributed to the fact that they are commensals of the bowels and that infections are mostly by fecal contamination due to poor hygiene and the presence of unique structure, which promote colonization of the host epithelial cells within the urinary tract and prevent bacteria from urinary washing [21].

In this study, higher resistance of *E. coli* was seen to tetracycline (60%). This was relatively comparable with a study conducted in Nekemte, Ethiopia (80.6%) [13], and Gondar, Ethiopia (80.2%) [11]. The higher resistance in *S. aureus* to tetracycline (85.7%) and nitrofurantoin (85.7%) was in line with a study conducted in Gondar, Ethiopia [11], and Hawassa, Ethiopia [14]. The similarity might be attributed to poor adherence to antibiotics, the use of antibiotics as a prophylactic treatment, easy availability, and indiscriminate use of antimicrobials by health professionals and patients.

In the current study, the odds of UTI were found to be 3.6 times higher among diabetic females than males. This was in accordance with previous studies conducted in Debre Tabor, Northwest Ethiopia [12], United States of America [22], and Timisoara, Romania [23]. The higher risk of infection in females might be due to the fact that urethra in females is much shorter and very close to the anus, which is a persistent source of fecal bacteria irrespective of DM [13, 24].

In the present study, the odds of UTI were 2.6 times higher among diabetic patients who were not able to read and write than those who able to read and write. This was consistent with a study conducted in Nekemte, Ethiopia [13]. The possible explanation for this similarity might be explained in terms of the low level of awareness on how to keep personal hygiene [8].

In this study, the odds of developing UTI were significantly higher for diabetic patients who had a history of UTI than for those who had no history of UTI. This was consistent with previous studies conducted in Nekemte, Ethiopia [13], but it was contradicted with a study conducted in Hawassa, Ethiopia [14], which showed a diabetic patient with no previous history of UTI had higher odds of UTI. The possible explanation for the difference might be due to relapse of the infection as a result of ineffective treatment and the presence of high concentration of sugar in diabetic urine, which serves as a media for a proliferation of pathogenic bacteria, or recall bias [6, 7].

## 5. Conclusions

In this study, the prevalence of UTI among diabetic patients was relatively comparable with the previous studies conducted in Ethiopia. The most common isolates include *E. coli*, *Klebsiella* spp., and *S. aureus*. Gentamycin and amoxicillin-clavulanic acid are a drug of choice for the treatment of UTI. Tetracycline and norfloxacin should not be used for the treatment of UTI in diabetic patients. Isolation of uropathogenic bacteria and antimicrobial susceptibility testing are crucial for the treatment of UTI in diabetic patients.

## Figures and Tables

**Table 1:** Characteristics of the study participants attending Metu Karl Heinz Referral Hospital, Southwest Ethiopia, 2018.

Characteristics	No.	(%)
Sex		
Male	107	45.9
Female	126	54.1

Age group (in years)		
15–24	23	9.9
25–34	40	17.1
35–44	58	24.9
45–54	50	21.5
55–64	36	15.5
>64	26	11.1

Educational status		
Not able to read and write	60	25.8
Primary level (1–8^th^)	56	24.0
Secondary level (9–12^th^)	45	19.3
Tertiary level (>12^th^)	72	30.9

Occupational status		
Employed	94	40.3
Farmer	42	18.1
Merchant	15	6.4
Student	17	7.3
House wife	49	21.0
Daily labor	16	6.9

Monthly income (in USD)		
<36.6	139	59.7
36.6–72.3	18	7.7
>72.3	76	32.6

**Table 2:** Uropathogenic bacteria isolated from urine specimen taken from the study participants attending Metu Karl Heinz Referral Hospital, Southwest Ethiopia, 2018.

Bacterial isolates	No.	(%)
*E. coli*	10	25.6
*Klebsiella* spp.	8	20.5
*S. aureus*	7	17.9
CoNS	6	15.4
*P. aeruginosa*	4	10.3
*Proteus* spp.	3	7.7
*Enterococcus* spp.	1	2.6

**Table 3:** In vitro antimicrobial susceptibility pattern of Gram-negative bacteria from the urine of adult diabetic patients attending Metu Karl Heinz Referral Hospital, Southwest Ethiopia, 2018.

Antimicrobials	Pattern	*E. coli* (*n* = 10)	*Klebsiella* spp. (*n* = 8)	*Proteus* spp. (*n* = 3)	*P. aeruginosa* (*n* = 4)	Total (*n* = 25)
Amoxicillin-clavulanic acid	S	5 (50)	5 (62.5)	3 (100)	0 (0)	16 (64)
	I	2 (20)	2 (25)	0 (0)	0 (0)	4 (16)
	R	3 (30)	1 (12.5)	0 (0)	4 (100)	5 (20)

Ceftriaxone	S	8 (80)	7 (87.5)	3 (100)	4 (100)	21 (84)
	R	2 (20)	1 (12.5)	0 (0)	0 (0)	4 (16)

Ciprofloxacin	S	7 (70)	5 (62.5)	1 (33.3)	0 (0)	13 (52)
	R	3 (30)	3 (37.5)	2 (66.7)	4 (100)	12 (48)

Tetracycline	S	4 (40)	2 (25)	1 (33.3)	0 (0)	8 (32)
	R	6 (60)	6 (75)	2 (66.7)	4 (100)	17 (68)

Gentamycin	S	7 (70)	7 (87.5)	1 (33.3)	2 (50)	17 (68)
	I	1 (10)	0 (0)	0 (0)	2 (50)	3 (12)
	R	2 (20)	1 (12.5)	2 (66.7)	0 (0)	5 (20)

Nitrofurantoin	S	6 (60)	6 (75)	2 (66.7)	0 (0)	14 (56)
	I	1 (10)	0 (0)	1 (33.3)	0 (0)	2 (8)
	R	3 (30)	2 (25)	0 (0)	4 (100)	9 (36)

Norfloxacin	S	6 (60)	5 (62.5)	1 (33.3)	0 (0)	12 (48)
	R	4 (40)	3 (37.5)	2 (66.7)	4 (100)	13 (52)

**Table 4:** In vitro antimicrobial susceptibility pattern of Gram-positive bacteria from the urine of adult diabetic patients attending Metu Karl Heinz Referral Hospital, Southwest Ethiopia, 2018.

Antimicrobials	Pattern	*S. aureus* (*n* = 7)	CoNS (*n* = 6)	*Enterococcus* spp. (*n* = 1)	Total (*n* = 14)
Ampicillin	S	0 (0)	3 (50)	1 (100)	4 (28.6)
	I	2 (28.6)	1 (16.7)	0 (0)	3 (21.4)
	R	5 (71.4)	2 (33.3)	0 (0)	7 (50)

Amoxicillin-clavulanic acid	S	6 (85.7)	6 (100)	1 (100)	13 (92.8)
	R	1 (14.3)	0 (0)	0 (0)	1 (7.1)

Ceftriaxone	S	1 (14.3)	4 (66.7)	1 (100)	6 (42.9)
	I	2 (28.6)	0 (0)	0 (0)	2 (14.2)
	R	4 (57.1)	2 (33.3)	0 (0)	6 (42.9)

Ciprofloxacin	S	3 (42.9)	2 (33.3)	1 (100)	6 (42.9)
	I	0 (0)	1 (16.7)	0 (0)	1 (7.1)
	R	4 (57.1)	3 (50)	0 (0)	7 (50)

Tetracycline	S	1 (14.3)	2 (33.3)	0 (0)	3 (21.4)
	I	0 (0)	1 (16.7)	0 (0)	1 (7.2)
	R	6 (85.7)	3 (50)	1 (100)	10 (71.4)

Gentamycin	S	4 (57.1)	5 (83.3)	1 (100)	10 (71.4)
	I	3 (42.9)	0 (0)	0 (0)	3 (21.4)
	R	0 (0)	1 (16.7)	0 (0)	1 (7.2)

Nitrofurantoin	S	0 (0)	1 (16.7)	0 (0)	1 (7.1)
	I	1 (14.3)	2 (33.3)	1 (100)	4 (28.6)
	R	6 (85.7)	3 (50)	0 (0)	9 (64.3)

Norfloxacin	S	3 (42.9)	3 (50)	1 (100)	7 (50)
	R	4 (57.1)	3 (50)	0 (0)	6 (50)

**Table 5:** Bivariate and multivariate regression analysis of factors associated with UTI among adult diabetic patient attending Metu Karl Heinz Referral Hospital, Southwest Ethiopia, 2018.

Patient characteristics	UTI		COR (95% CI)	AOR (95% CI)
	Positive no (%)	Negative no (%)		
Sex				
Male	7 (6.5)	100 (93.5)	1	1
Female	32 (25.4)	94 (74.6)	4.86 (2.05, 11.55)	3.56 (1.44, 8.76)

Age (in years)				
<35 years	12 (14.8)	69 (85.2)	1	
≥35 years	27 (17.8)	125 (82.2)	1.24 (0.59, 2.60)	

Educational status				
Not able to read and write	20 (11.6)	153 (88.4)	1	1
Able to read and write	19 (31.7)	41 (68.3)	3.54 (1.73, 7.26)	2.55 (1.19, 5.49)

Use of antibiotics				
Yes	13 (14.6)	76 (85.4)	1.29 (0.62, 2.66)	
No	26 (18.1)	118 (81.9)	1	

History of UTI				
No	15 (11)	121 (89)	1	1
Yes	24 (24.7)	73 (75.3)	2.65 (1.31, 5.38)	2.31 (1.09, 4.90)

Current symptom of UTI				
No	30 (14.8)	173 (85.2)	1	1
Yes	9 (30)	21 (70)	2.47 (1.03, 5.91)	2.00 (0.78, 5.13)

Type of diabetes				
Type II	19 (14)	116 (86)	1	1
Type I	20 (20.4)	78 (79.6)	1.56 (0.78, 3.12)	1.430 (0.67, 3.03)

History of hypertension				
No	21 (17.8)	97 (82.2)	1	
Yes	18 (15.7)	97 (84.3)	0.86 (0.43, 1.71)	

Duration of diabetes				
≤5 years	21 (17.4)	100 (82.6)	1	
>5 years	18 (16.1)	94 (83.9)	0.91 (0.46, 1.82)	

History of glucosuria				
No	18 (13.2)	118 (86.8)	1	1
Yes	21 (21.6)	76 (78.4)	1.81 (0.91, 3.62)	1.53 (0.73, 3.23)

Types of DM medication				
Oral	20 (19.4)	83 (80.6)	1	
Injection	19 (14.6)	111 (85.4)	0.71 (0.36, 1.41)	

COR: crude odds ratio; AOR: adjusted odds ratio; CI: confidence interval.

## Data Availability

The data used to support the findings of this study are included within the article and are available (the SPSS data) from the corresponding author upon request.
